# e-Learning development in medical physics and engineering

**DOI:** 10.2349/biij.4.1.e27

**Published:** 2008-01-01

**Authors:** S Tabakov

**Affiliations:** Department of Medical Engineering and Physics, King's College Hospital, London, United Kingdom

**Keywords:** Medical Physics, e-Learning, education

## Abstract

Medical Physics and Engineering was among the first professions to develop and apply e-Learning (e-L). The profession provides excellent background for application of simulations and other e-L materials. The paper describes several layers for e-L development: Programming specific simulations; Building e-L modules; Development of e-L web-based programmes. The paper shows examples from these layers and outlines their specificities. At the end, the newest e-L development (project EMITEL) is briefly introduced and the necessity of a regularly updated list of e-L activities is emphasised.

## INTRODUCTION

Medical Physics and Engineering (MEP) was among the first professions to develop and apply e-Learning (e-L). An indicator for this is the first international prize in the field (EU Leonardo da Vinci Award) presented to European Medical Imaging Technology (EMIT) Consortium in 2004.

During the last 15 years, a number of activities and publications addressed the questions of MEP Education and Training [1, 2, 3, 4]. These led to rapid development of the profession worldwide and now the next stage of e-L is to be addressed. A special issue on the subject was published by the Journal of Medical Engineering and Physics in 2005 [5]. Based on the paper and on the authors’ 12 years’ experience in e-L, the following key-elements can be specified for the introduction and use of e-L in MEP:

1. e-L is imperative for MEP because it offers quick and easy update of teaching materials – a very important function for this dynamic profession. This, combined with the fast delivery of the content through Internet, makes e-L materials the first choice for many lecturers;
2. e-L proposes an elegant way to solve the problem through the understanding of complex physics models. Using interactive simulations, computer diagrams or images leads to increased effectiveness of the learning process.
3. Images are specifically important for MEP and e-L provides a cheap and effective means for publishing large number of images (either on CD or through the Web). Additionally e-L can offer a means of image manipulation, which has no analogue in other means of publications.
4. The SEARCH function offered by various e-L materials is another important advantage. This is also imperative for MEP, where currently the specific terms number more than 3,000. This was specially used in the EMITEL Dictionary and Encyclopedia.
5. Finally, the fact that many students from around the world can use the materials through the guidance of most renowned specialists has no analogue in the other educational methods and media.

## E-L DEFINITIONS AND LAYERS OF DEVELOPMENT

The definitions of e-Learning vary significantly, but perhaps “… learning that is aided by information and communication technologies…” [7] is one of the definitions, which is very close to reality. However, some authors and users define e-L only as “… the delivery of content via all electronic media, including the internet, intranets, extranets, satellite, broadcast, video, interactive TV, and CD ROM…”[6]. In this case the emphasis is only at the delivery and many unworthy courses have been “developed” this way – by just delivery to students of existing files with handouts. This has the only advantage of reduced cost, but the educational results do not have significant value.

The authors certainly support the first view/definition – that e-L should develop materials, which will increase the pedagogical effectiveness, and then deliver these to students. Only in this case the full power of e-L can be utilised.

The development of e-L materials can be presented as a multi-layered process, including the following stages:

- programming specific simulations;
- building of e-L modules;
- development of e-L programmes.

These stages most often exist as separate entities, but the programmes will include modules, and simulations can be applied at various stages.

Further below this paper shall present some examples of various types of e-L materials. It is impossible to list (or even mention) all existing e-L activities, as this is a very dynamic area and a number of simulations constantly appear or disappear.

## E-L SIMULATIONS

The simulations are a very effective teaching tool. However, these are very difficult to produce and require good software skills. Also, development of simulations consumes a lot of time. This often requires knowledge of several software packs and skills to present the simulation in a suitable, pedagogical way. One specific problem with simulations is their relatively short life cycle (often less than 5 years). The main reason for this is the upgrade of software platform, what in most cases hampers the performance of the simulation. There is a need for quick dissemination of simulation tools, which could be made through a regularly updated list of available simulations.

Among the earliest developments in this field include the Gamma Camera DOS-simulator learning pack (developed some 15 years ago with the support of International Atomic Energy Agency (IAEA); the PowerLab Systems of AD Instruments; various LabView simulations; the IPEM X-ray Spectrum Processor software, etc. Other existing simulations are described in the special issue on e-Learning of JMEP [5].

Other examples of simulations can be found at the web sites of some manufacturers and professional societies such as AAPM, IPEM, etc. However, the profession lacks a comprehensive list of such tools, which would be of great value for education and training.

## E-L EDUCATIONAL MODULES

Building e-L educational modules is probably the most efficient e-L approach. This approach is used in a number of projects and Universities. In principle, one module could cover either educational or training needs (or both) and could have a length of several tens of hours (or days). This allows flexibility by merging the module (or parts of the module) with other pedagogical activities. In this way, the modules are best suited for hybrid delivery of education (classical plus e-L). Due to this reason, most e-L modules include an option for printing the materials.

Most e-L educational modules include only limited interactivity. Perhaps this is due to two reasons: reduced cost for development and increased life cycle. However, these include many images, hyperlinked with text, which increases significantly their educational effectiveness. Also, such modules are very convenient as e-books. Perhaps this is one of the reasons that almost all of them work without tools for students’ assessment.

e-L modules also allow flexible application at various levels (radiographers, radiologists, engineers and physicists). An example for such module is Demystifying Biomedical Signals – a module developed in the Southampton University [8].

Some modules include mainly PowerPoint materials and HTML web pages, but are of extreme importance for the professions. The sets of presentations of the Sprawls Educational Foundation (www.sprawls.org) and of the IAEA (web site on Radiological Protection of the Patient) deserve special mention. The IAEA web site includes materials on Protection in: Radiotherapy, Nuclear Medicine and Diagnostic Radiology (with special emphasis on Interventional Radiology) [9]. These materials (mainly the PowerPoint slides) have been additionally disseminated on CDs to almost all IAEA member states and have been widely used for educational/training courses at various levels.

Typical examples for e-L modules are also the EMERALD and EMIT materials (based on EU projects headed by King’s College London and King’s College Hospital) [10, 11]. These include five volumes (on five CDs and on web site). The first three volumes on EMERALD cover Physics and Equipment of X-ray diagnostic radiology, Nuclear medicine and Radiotherapy. The next two volumes on EMIT cover Physics and Equipment of Ultrasound and Magnetic Resonance Imaging. These modules are currently used in more than 60 countries, either as training materials or in University Labs. Each of these original e-L module incorporates: List of Competencies (in accordance with the UK’s IPEM Training scheme); Structured Timetable (training curricula); Student Workbook with practical training tasks; and Image database (to support the practical training tasks).

**Figure 1:**
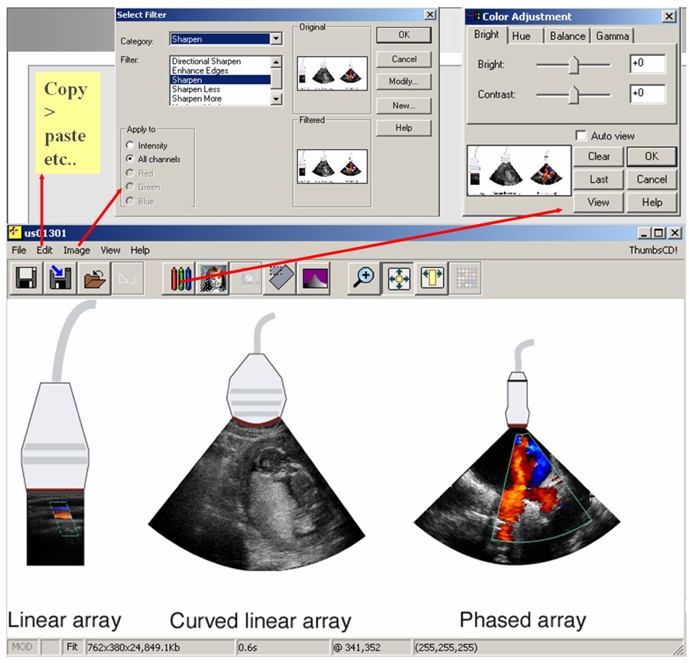
Graphical interface (print-screen) of the EMIT Image Database CD-ROM with ThumbPlus! browser - sample from Module Ultrasound Imaging, with some image processing functions.

EMERALD and EMIT were the first modules to introduce the concept of Educational Image databases and e-books. A large demo of the e-books is available at the web site www.emerald2.eu. The software of the Image database allows interactive off-line manipulation of images (Figure 1).

## E-L STUDY PROGRAMMES

Structuring e-L study programmes is the most complex e-L activity, however it is not always the most innovative of all. Many Universities are now transferring their existing classical courses to web-platforms for Virtual Learning Environment (VLE) like WebCT, Blackboard, etc. These platforms allow easy online administration of the course, but do not always provide new updated teaching materials. It is believed that this will change in future. Good examples are the courses in the University of Adelaide, Australia and the Vanderbilt University, Nashville, USA [12,13].

Other web-based courses have orientated themselves to the development of specific web-platforms. This approach is very difficult and expensive, but allows the inclusion of custom-built materials and simulations alongside the administration tools of the programme. Attempts in this line have been made, among others, in the University of Gratz, Austria, and Medical University, Plovdiv, Bulgaria [14,15]. These programmes are discussed in detail in the special issue on e-L of JMEP.

A typical feature of these activities is that the web-based programme can be applied in one University only (usually these are password-protected with access for local students). Another feature is that these programmes are difficult to maintain. Normally the web site would require constant support from a dedicated experienced webmaster. Due to the recent initiation of a large number of such programmes, an EU project was recently launched (headed by Ragnar Granit Institute at Tampere University of Technology, Finland). This project (European Virtual Campus for Biomedical Engineering - EViCaB) [16] is related primarily to Medical Engineering, but could extend (or provide information) also for the Medical Physics programmes.

## EVALUATION OF E-L MATERIALS

The usefulness of e-L materials has not been disputed, but these require careful assessment. The first Conference to address e-L in Medical Physics was held at International Centre for Theoretical Physics (ICTP), Trieste, during November 2003. Specialists from 26 countries gathered at this Conference to discuss the EMIT e-L materials and the first feedback from the users.

Two independent surveys were made at this stage to assess separate tasks on X-ray Diagnostic Radiology and on Ultrasound Imaging. The supervisors reported that in both surveys the students felt better prepared after the e-L task (compared with the classical training) and required less time to complete the task (in average 20-40% less time, student dependent). The students also reported 25-35% improvement in their knowledge after using the e-L tasks, which indicates that the material had been effectively used [17].

Most of the delegates at this Conference agreed on the following main issues related to e-L:

- e-L is not only suitable, but essential for a dynamic profession like Medical Physics and Engineering;
- e-L increases enormously the effectiveness of knowledge transfer, but needs to be combined with classical learning – HYBRID learning;
- The software platform is crucial for the life cycle of the e-L products (sometimes only an upgrade of the software version can hamper the e-L product);
- The e-L development team is multi-professional, requiring good professional knowledge, pedagogical experience and IT skills;
- Producing e-L content is very difficult and costly;
- e-L development is not less innovative than any other research;
- The efforts to produce e-Learning materials are often underestimated by students and Universities;
- There is a need of sound multi-professional forum on e-L.

## NEWEST DEVELOPMENTS

The latest development in e-L is the European Medical Imaging Technology e-Encyclopaedia for Lifelong Learning (EMITEL) project, which developed the first e-L multilingual e-Dictionary of Medical Physics and the first e-Encyclopedia of Medical Physics. This large international project (headed by King’s College London and King’s College Hospital) includes more than 90 professionals and the first results (the e-Dictionary) are in very advanced stage (www.emitdictionary.co.uk). It cross-translates Medical Physics terms to/from any of its 15 languages and is soon to be updated to more than 20 languages. This activity will allow colleagues from all over the world to use effectively the existing teaching materials (most of which are in English).

The EMITEL e-Encyclopedia is expected to be launched during the World Congress of Medical Physics and Bioengineering (Munich, 2009). It will explain all terms from the Dictionary with articles (including text, images and diagrams). Its volume is expected to grow up to above 5 GB. A special Conference will discuss these issues by the end of 2008.

**Figure 2:**
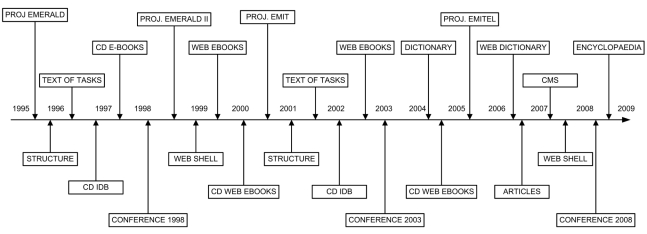
Timeline of four interlinked e-L projects, showing the progress from the first pilot project (1995) to the e-Encyclopaedia project (to be completed in 2009). The timeline is presented in 3 layers - interim results (most inner layer), final results (middle layer), project development and assessment (Conferences) - most outer layer.

EMITEL is based on the results from the previous e-L projects EMERALD, EMERALD II and EMIT. A timeline of their development shows the main stages of these projects (Figure 2). It is clear that the ideas developed in one project had been carried over in the next one. Similarly materials from the first projects had been updated incorporated in the last project EMITEL, thus increasing the efficiency of the e-L production. The timeline shows the sequence of stages, but can not show the time of their development, as it varies from approximately 3 to 18 months

## CONCLUSION

e-Learning has already found a steady presence in Medical Engineering and Physics. A large number of simulations, e-L modules and web-based programmes have been developed and implemented in practice. Some of those, such as the AAPM Virtual Library, the IAEA Training materials and web sites, the EMERALD and EMIT, etc., have reached wide audiences. Many of those materials are still not known therefore these activities need to be urgently listed in order to allow effective exchange of knowledge. Some attempts have been made in this direction – for example, the book Internet for Radiology, the Global On-Line Medical Physics (GOMP), EMITEL Encyclopedia, several publications, etc. [18, 19, 20]. However, the effective and wide use of e-L will be stabilised only if a good and updated list of these materials is available free for all professionals.
